# Optimal Duration of Compression Stocking Therapy after Endovenous Laser Ablation Using a 1470-nm Diode Dual-Ring Radial Laser Fiber for Great Saphenous Vein Insufficiency

**DOI:** 10.3400/avd.oa.21-00012

**Published:** 2021-06-25

**Authors:** Shinsuke Mii, Atsushi Guntani, Ryosuke Yoshiga, Takuya Matsumoto, Eisuke Kawakubo, Jun Okadome

**Affiliations:** 1 Department of Vascular Surgery, Saiseikai Yahata General Hospital, Kitakyushu, Fukuoka, Japan; 2 Department of Surgery, Saiseikai Fukuoka General Hospital, Fukuoka, Fukuoka, Japan

**Keywords:** endovenous laser ablation, compression therapy, elastic stocking, high-wavelength laser

## Abstract

**Objective:** To investigate the optimal duration of compression therapy after endovenous laser ablation (EVLA) using a 1470-nm diode dual-ring radial laser fiber for great saphenous vein (GSV) insufficiency.

**Methods:** Patients undergoing EVLA of GSV for varicose vein disease were divided into two groups based on the duration of subsequent compression after the procedure: short duration group (S group; 0–2 days) and long duration group (L group; 1–4 weeks). Patient-reported outcomes (pain and quality of life [QOL]) were set as the primary outcomes, and objective findings (venous clinical severity score [VCSS], leg circumference, and duplex ultrasound [DUS] findings) were set as the secondary outcomes. A follow-up examination was performed at 1 week and 1 and 6 months. Each variable between the groups was compared after a propensity score matching using the age, sex, Clinical–Etiological–Anatomical–Pathophysiological (CEAP) clinical class, job type, and target variable as covariates. A per-protocol analysis was performed.

**Results:** The S and L groups included 98 and 99 patients, respectively. A propensity score matching analysis showed no significant differences between the groups in any outcomes at any follow-up intervals.

**Conclusion:** Long-term compression showed little benefit; therefore, the prescription of compression stocking beyond 2 days after EVLA is unnecessary.

## Introduction

The prescription of compression stockings after intervention for varicose vein disease is a standard practice; however, the necessity and optimal duration of compression remains controversial.^1–4)^ Regarding the optimal duration of compression after endovenous thermal ablation (ETA), no current guidelines have described a clear-cut recommendation because of insufficient evidence.^1,3,4)^

Several prospective studies using low-wavelength (810 nm) lasers demonstrated that a longer duration (≥1 week) of compression was superior to a shorter duration mainly because of less pain at 1 week after the procedure.^5–7)^ However, these results are not informative in the current clinical setting because high-wavelength lasers are mainly used.

In Japan, laser system of 980-nm and 1470-nm wavelength were launched in 2011 and 2014, respectively. The first guideline regarding endovascular treatment in Japan, published in 2010, recommended daytime compression for 1–4 weeks following whole-day compression for several days.^8)^ This recommendation was also included in the new guideline released in 2019.^4)^ However, we know from experience that a 1470-nm diode dual-ring radial laser fiber produces less pain at the treated vein, as several authors have indicated.^9–11)^ Thus, we hypothesize that a shorter duration (≦2 days) of the additional compression is sufficient when endovenous laser ablation (EVLA) is performed using a 1470-nm diode dual-ring radial laser fiber.

The present study explored the optimal duration of compression after EVLA using a 1470-nm diode dual-ring radial fiber laser.

## Patients and Methods

The institutional review board of Saiseikai Yahata General Hospital, Kitakyushu City, Japan, approved the study design (approval number: 82). The informed consent of all patients was obtained when they decided the date of EVLA.

### Study design

Adult patients who underwent EVLA of the great saphenous vein (GSV) for varicose vein disease, estimated as Clinical–Etiological–Anatomical–Pathophysiological (CEAP) clinical class 2–4, in a unilateral limb were enrolled between January 2016 and December 2018. All GSVs undergoing EVLA had the condition of a reflux of >0.5 seconds at the saphenofemoral junction (SFJ) or an incompetent perforating vein of the femoral canal on a duplex ultrasound (DUS). GSVs >20 mm in diameter were excluded.

Patients were divided into two groups based on the duration of compression after EVLA: the short duration group (S group) with no compression or two days of compression and the long duration group (L group) with 1–4 weeks of compression. Patients decided the duration of compression by themselves after receiving an explanation that the Japanese guideline recommends at least 1 week of compression.^8)^ The exclusion criteria were age of <20 years old, CEAP clinical class 5 or 6, simultaneous ablation of multiple saphenous veins or simultaneous intervention in the bilateral limbs, and a history of treatment for varicose veins within 6 months.

Before admission, data on patients’ background characteristics (age, sex, job that involved standing, CEAP clinical class), clinical findings (venous clinical severity score [VCSS], leg circumference at the mid-calf and ankle), DUS findings (diameter of the GSV at 5 cm distal to SFJ, mid-thigh, and upper edge of the patella; reflux time of the SFJ; presence of Dodd’s incompetence perforator; varicosity at the SFJ), pain (visual analog pain scale [VAS], use of analgesics for chronic pain), and quality of life (QOL; 20-item Chronic Venous Disease Quality of Life Questionnaire [CIVIQ-20],^12,13)^ MOS 36-Item Short-Form Health Survey [SF-36]^14,15)^) were collected. SF-36 was calculated based on the national standard values.

As operative information, the operative time, total energy, ablation time, ablation length, total volume of tumescent local anesthesia (TLA), and number of stab avulsions were recorded. The use of analgesics after EVLA during hospitalization and VAS at discharge were also investigated.

### Outcomes

The primary outcomes were patient-reported outcomes, such as the pain (VAS, use of analgesics) and QOL (CIVIQ-20, SF-36), which were checked by patients themselves after the procedure on marking was explained by nurses. The secondary outcomes were objective outcomes, such as clinical findings (VCSS, leg circumference) and DUS findings (recanalization of the ablated GSV, deep vein thrombosis [DVT]). VCSS was checked by a doctor who performed EVLA, and compressive therapy in VCSS was deemed to be absent in all patients at any examination points. The period of off-work for patients who had a regular job was also investigated.

A follow-up examination was performed at 1 week and 1 and 6 months. Additional treatment for varicose vein disease in either limb was inhibited until the end of the study.

### Flow from admission to discharge

Patients underwent EVLA using a 1470-nm ELVes Radial 2ring Fiber® (Integral Corp., Tokyo, Japan) under general and TLA anesthesia on the day of admission. The GSV was ablated from 1 to 2 cm distal to the SFJ to the first 3–4 cm with a linear endovascular energy density (LEED) of 100 J/cm and subsequently ablated to the puncture site around the knee with LEED of 50 J/cm. Stab avulsion for varices of ≥4 mm in diameter was added. After the procedure, a long gauze, rolled into a tubular form, was placed along the ablated vein, and elastic bandages were wrapped around the whole leg. The patients spent the night in the hospital. The next morning, they were discharged after their operative wounds were checked and a compression stocking had been pulled on (thigh-high, graduated type; 20 mmHg at the ankle; Ansilk II®, ALCARE Co., Ltd., Tokyo, Japan) if they were part of a compression group.

### Analyses

A per-protocol analysis was performed. Patients who changed the duration of compression from the S group to the L group or from the L group to the S group after EVLA were excluded. Patients who changed the duration of compression in the same group were not excluded. Analyses were performed for the whole cohort and after a propensity score matching. The age, sex, CEAP clinical class, presence of a standing job, and preoperative data of the target variables were used as covariates for matching. The postoperative use of analgesics was investigated only in patients who did not take analgesics for chronic pain before admission.

### Definitions

Recanalization was defined as the flow signal of the whole ablated GSV on the DUS. The presence of a job requiring standing was determined according to the patient report. The duration of off-work was defined as the number of days from EVLA to restarting work.

### Statistical analyses

Data for categorical variables are presented as the number (percentage). Data for continuous variables with and without a normal distribution are presented as the mean±standard deviation and the median (interquartile range), respectively.

Categorical variables were compared using the Fisher’s exact test or Pearson’s test, and continuous variables with and without a normal distribution were compared using an unpaired Student’s t-test and the Wilcoxon’s rank-sum test in the whole cohort, respectively. In a propensity score matching analysis, categorical variables were compared using the McNemar’s test, and continuous variables with and without a normal distribution were compared using a paired Student’s t-test and the Wilcoxon’s signed-rank test, respectively. The propensity score matching analysis was performed using a 1 : 1 matching protocol without replacement (Greedy matching algorithm) with a caliper width equal to 0.25 of the standard deviation of the logit of the propensity score.

P values of <0.05 were considered to indicate statistical significance. The statistical analyses were performed using the JMP software program version 14.2 for Mac (SAS Institute, Cary, NC, USA).

## Results

### Patient flow in the analysis

During the period for enrollment, 218 patients underwent EVLA of the GSV for varicose vein disease in a unilateral limb. Five patients were excluded because they did not meet the inclusion criteria: CEAP clinical class 5 or 6 (n=3), <20 years old (n=1), and rejection of participation (n=1). Accordingly, a total of 213 patients were enrolled in this study.

**Figure 1:**
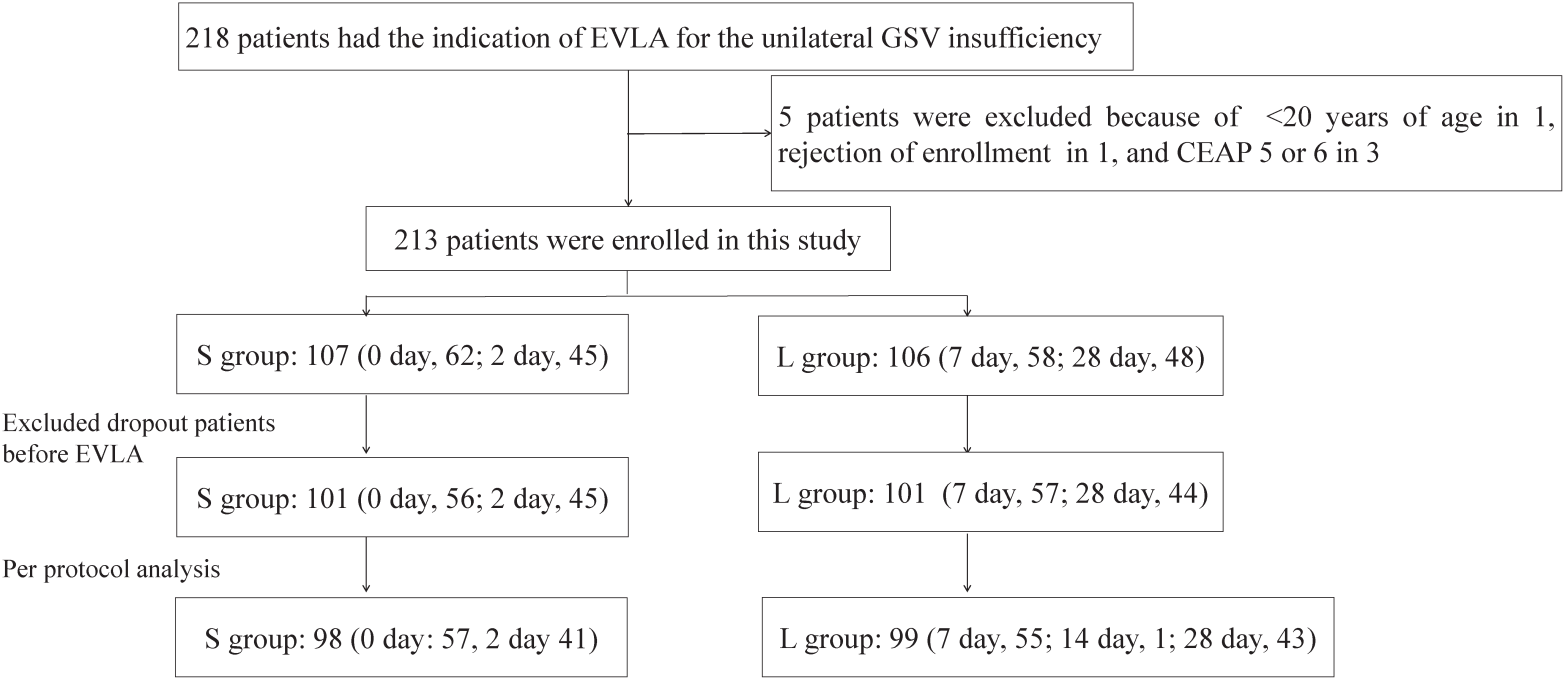
Fig. 1 The patient-flow for per-protocol analysis. Details regarding the dropout patients before EVLA and change of the group are described in the text.

EVLA: endovenous laser ablation; GSV: great saphenous vein; CEAP: Clinical–Etiological–Anatomical–Pathophysiological clinical class

During the period between the enrollment and admission, another 11 patients cancelled their planned EVLA procedure because of the patient’s request (n=5); accident injury (n=2); and operation for spinal cord stenosis, onset of DVT, a diagnosis of moderate arterial valve stenosis, and thrombosis of GSV (n=1 each). Accordingly, 202 patients (101 patients in each group) underwent EVLA. After EVLA, three patients from the S group and two patients from the L group changed the duration of compression to ≥1 week and ≤2 days, respectively. Consequently, 197 patients were included in the present analysis. The S and L groups included 98 and 99 patients, respectively (**Fig. 1**).

### Baseline characteristics

#### Preoperative data

Patients’ background data, including VCSS, leg circumference, and the information regarding pain and QOL before EVLA, are shown in **Table 1**. There were no significant differences between the groups, except for the proportion with a standing job (S group, 17%; L group, 31%; p=0.031) and the domain of mental health of SF-36 (S group, 50.3±9.6; L group, 46.6±10.3; p=0.009).

**Table 1:** Table 1 Preoperative data

	S (n=98)	L (n=99)	p
Age (years)	68.4±10.5	67.0±11.7	0.385
Sex, female	63/98, 64%	66/99, 67%	0.766
Laterality, left	55/98, 56%	51/99, 52%	0.569
CEAP2/3/4	48/27/23	33/37/29	0.081
Standing job	17/98, 17%	31/99, 31%	**0.031**
VCSS	2.9±1.6 (n=97)	3.1±1.9 (n=96)	0.343
Leg circumference			
Mid-calf (cm)	36.4±3.4 (n=90)	36.0±3.1 (n=94)	0.487
Ankle (cm)	22.3±2.1 (n=92)	21.9±1.7 (n=93)	0.177
Pain			
VAS	2.9±2.7 (n=98)	3.0±2.6 (n=99)	0.872
Use of analgesics	11/97, 11%	17/97, 18%	0.307
QOL			
CIVIQ-20	73.2±16.1 (n=96)	72.0±18.8 (n=97)	0.643
SF-36			
PF_N	41.1±15.1 (n=98)	40.3±13.6 (n=98)	0.718
RP_N	44.9±13.3 (n=98)	42.1±12.7 (n=99)	0.125
BP_N	46.8±10.8 (n=98)	46.3±11.4 (n=99)	0.721
GH_N	45.0±8.1 (n=98)	47.1±8.4 (n=98)	0.445
VT_N	49.9±9.9 (n=98)	47.3±10.8 (n=98)	0.086
SF_N	49.2±10.3 (n=98)	46.7±11.9 (n=99)	0.105
RE_N	47.2±12.1 (n=98)	43.9±12.9 (n=99)	0.063
MH_N	50.3±9.6 (n=98)	46.6±10.3 (n=98)	**0.009**

CEAP: Clinical–Etiological–Anatomical–Pathophysiological clinical class; VCSS: venous clinical severity score; GSV: great saphenous vein; VAS: visual analog pain scale; QOL: quality of life; CIVIQ-20: 20-item Chronic Venous Disease Quality of Life Questionnaire; SF-36: MOS 36-Item Short-Form Health Survey; PF_N: physical functioning (based on national standard value); RP: role physical; BP: bodily pain; GH: general health; VT: vitality; SF: social functioning; RE: role emotional; MH: mental health

#### Operative data

There were no significant differences between the S and L groups in the operative time (33.6±13.4 vs. 33.4±14.7 min; p=0.922), total energy (1813±389 vs. 1832±386 J; p=0.727), ablated GSV length (32.1±6.7 cm vs. 32.5±6.8 cm; p=0.638), ablation time (182±40 vs. 184±38 sec; p=0.791), total volume of TLA (304±109 vs. 307±112 ml; p=0.867), number of wounds due to stab avulsion (7.7±5.5 vs. 7.1±6.1; p=0.490), or the rate of patients with simultaneous stab avulsion (89.8% vs. 86.9%; p=0.658).

#### Inhospital data after EVLA

There were no significant differences between the groups in the VAS (1.7±2.0 vs. 1.9±1.9 in S group [n=92] and L group [n=89], respectively; p=0.614) at discharge or use of analgesics during hospitalization among patients who had not received analgesics for chronic pain before admission (5.8% vs. 8.8% in S group [n=86] and L group [n=80], respectively; p=0.555).

#### Follow-up data

Only the results of the propensity score matching analysis are described in the text. Those of the whole cohort are shown in **Table 2**.

**Table 2:** Table 2 Outcomes of the whole cohort

*Patient-reported outcomes*				
VAS				
		S group	L group	p
1 w		2.2±1.7 (n=97)	2.3±1.7 (n=99)	0.555
1 m		1.6±1.7 (n=95)	1.8±1.9 (n=99)	0.525
6 m		1.2±1.9 (n=86)	1.3±1.8 (n=92)	0.686
Use of analgesics				
		S group	L group	p
1 w		4, 4.8% (n=82)	9, 11.7% (n=77)	0.151
1 m		11, 13.6% (n=81)	10, 12.7% (n=79)	1.000
6 m		5, 6.9% (n=72)	5, 6.7% (n=75)	1.000
CIVIQ-20				
		S group	L group	p
1 w		74.5±16.2 (n=95)	74.9±17.2 (n=95)	0.855
1 m		84.1±15.3 (n=93)	81.8±18.4 (n=98)	0.341
6 m		85.5±15.4 (n=85)	84.0±19.1 (n=95)	0.578
SF-36				
		S group	L group	p
PF_N	1 w	36.5±16.2 (n=95)	38.2±15.7 (n=98)	0.455
	1 m	41.4±15.1 (n=93)	41.4±15.9 (n=98)	0.988
	6 m	41.6±15.8 (n=85)	41.9±17.8 (n=95)	0.893
RP_N	1 w	40.3±14.7 (n=95)	41.7±13.6 (n=98)	0.498
	1 m	46.1±11.7 (n=94)	45.4±13.7 (n=98)	0.709
	6 m	46.7±11.1 (n=85)	47.0±12.1 (n=95)	0.872
BP_N	1 w	46.2±9.9 (n=95)	47.0±9.4 (n=98)	0.548
	1 m	49.1±9.2 (n=94)	50.4±9.7 (n=98)	0.333
	6 m	51.3±10.7 (n=85)	52.0±9.7 (n=95)	0.614
GH_N	1 w	49.0±7.9 (n=95)	49.2±8.4 (n=98)	0.849
	1 m	49.7±8.2 (n=94)	48.6±9.1 (n=98)	0.385
	6 m	48.7±8.4 (n=85)	48.3±8.2 (n=95)	0.719
VT_N	1 w	49.9±10.5 (n=95)	50.0±9.9 (n=98)	0.950
	1 m	51.2±9.3 (n=94)	50.3±10.3 (n=98)	0.538
	6 m	50.8±10.4 (n=85)	49.8±10.2 (n=95)	0.509
SF_N	1 w	48.4±10.6 (n=95)	46.6±11.4 (n=98)	0.264
	1 m	49.3±10.9 (n=94)	48.3±11.2 (n=98)	0.532
	6 m	50.2±10.0 (n=85)	49.7±9.7 (n=95)	0.735
RE_N	1 w	44.3±13.8 (n=95)	43.7±13.0 (n=98)	0.735
	1 m	49.1±10.6 (n=94)	47.0±12.6 (n=98)	0.215
	6 m	49.0±10.9 (n=85)	47.1±12.2 (n=95)	0.269
MH_N	1 w	49.6±9.5 (n=95)	49.1±9.8 (n=98)	0.264
	1 m	51.6±9.2 (n=95)	50.8±9.2 (n=98)	0.526
	6 m	51.5±10.7 (n=85)	50.2±10.0 (n=95)	0.392
*Objective outcomes*				
VCSS				
		S group	L group	p
1 w		0.71±0.93 (n=92)	0.75±1.07 (n=97)	0.753
1 m		0.56±0.76 (n=91)	0.66±1.07 (n=95)	0.453
6 m		0.40±0.72 (n=86)	0.26±0.55 (n=95)	0.166
Leg circumference, % of the preoperative size				
		S group	L group	p
Mid-calf				
1 w (%)		97.4±3.8 (n=86)	97.3±4.2 (n=93)	0.829
1 m		96.7±3.7 (n=85)	96.8±4.3 (n=92)	0.854
6 m		97.3±4.0 (n=77)	96.9±4.7 (n=90)	0.486
Ankle				
1 w (%)		101.5±4.1 (n=88)	101.7± 6.0 (n=92)	0.798
1 m		101.6±4.4 (n=87)	101.0±6.3 (n=91)	0.443
6 m		101.5±5.2 (n=79)	100.5±4.8 (n=89)	0.177

VAS: visual analog pain scale; CIVIQ-20: 20-item Chronic Venous Disease Quality of Life Questionnaire; SF-36: MOS 36-Item Short-Form Health Survey; PF_N: physical functioning (based on national standard value); RP: role physical; BP: bodily pain; GH: general health; VT: vitality; SF: social functioning; RE: role emotional; MH: mental health; VCSS: venous clinical severity score

### Primary outcomes

#### Pain

**Figure 2:**
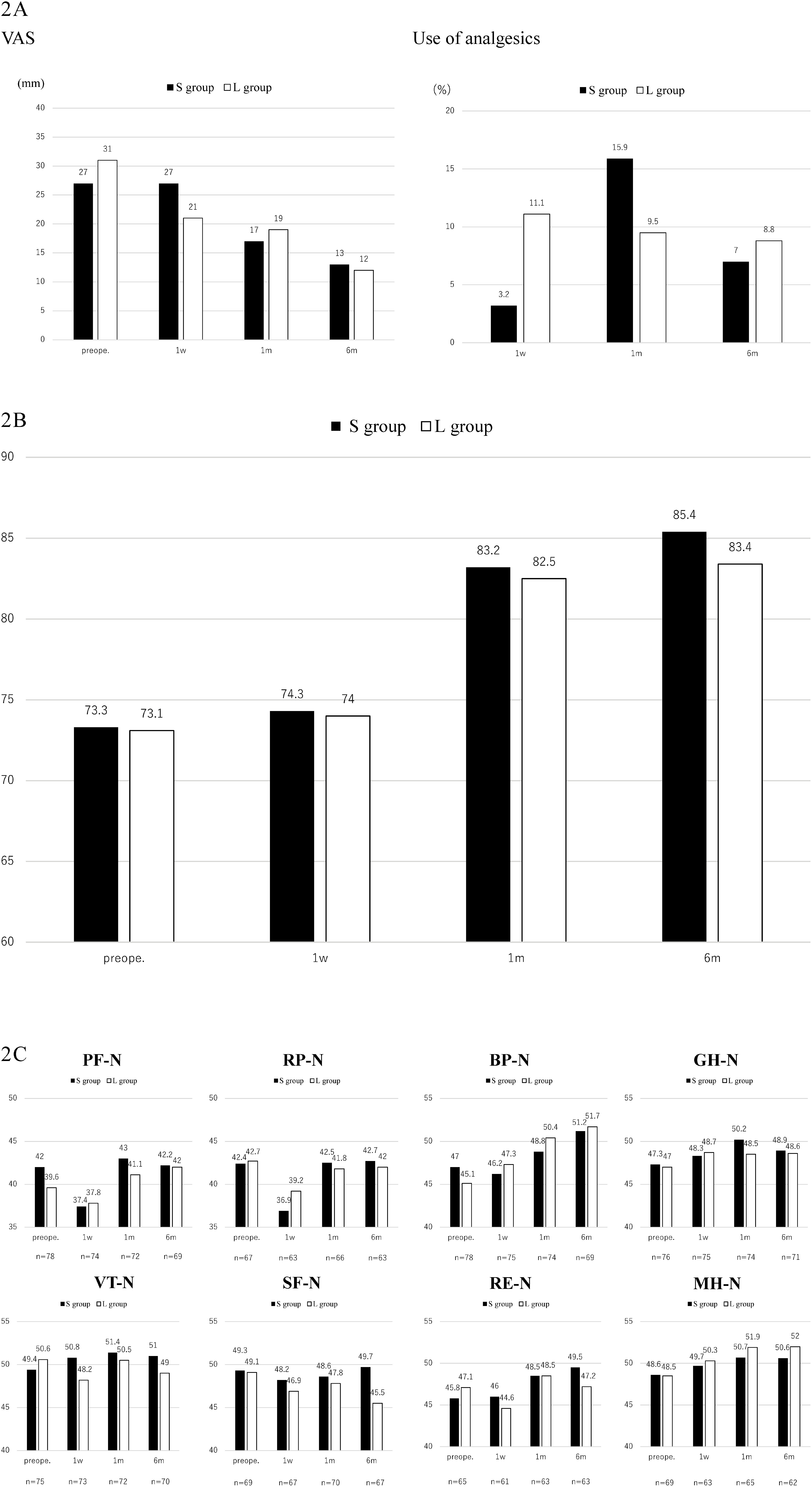
Fig. 2 Findings for primary outcomes based on a propensity score matching analysis. (**A**) VAS and use of analgesics. (**B**) CIVIQ-20. (**C**) SF-36.

VAS: visual analog scale; PF_N: physical functioning (based on national standard value); RP: role physical; BP: bodily pain; GH: general health; VT: vitality; SF: social functioning; RE: role emotional; MH: mental health

There were no significant differences between the groups at any intervals in the VAS, which decreased with time in both groups. There were also no significant differences between the groups in the use of analgesics (**Fig. 2A**).

#### QOL

The results of the CIVIQ-20 showed no significant differences between the groups at any intervals. The score increased with time and was markedly elevated at 1 and 6 months in both groups (**Fig. 2B**). Regarding the SF-36, there were no significant differences between the groups in any domains at any intervals (**Fig. 2C**).

### Secondary outcomes

#### VCSS

**Figure 3:**
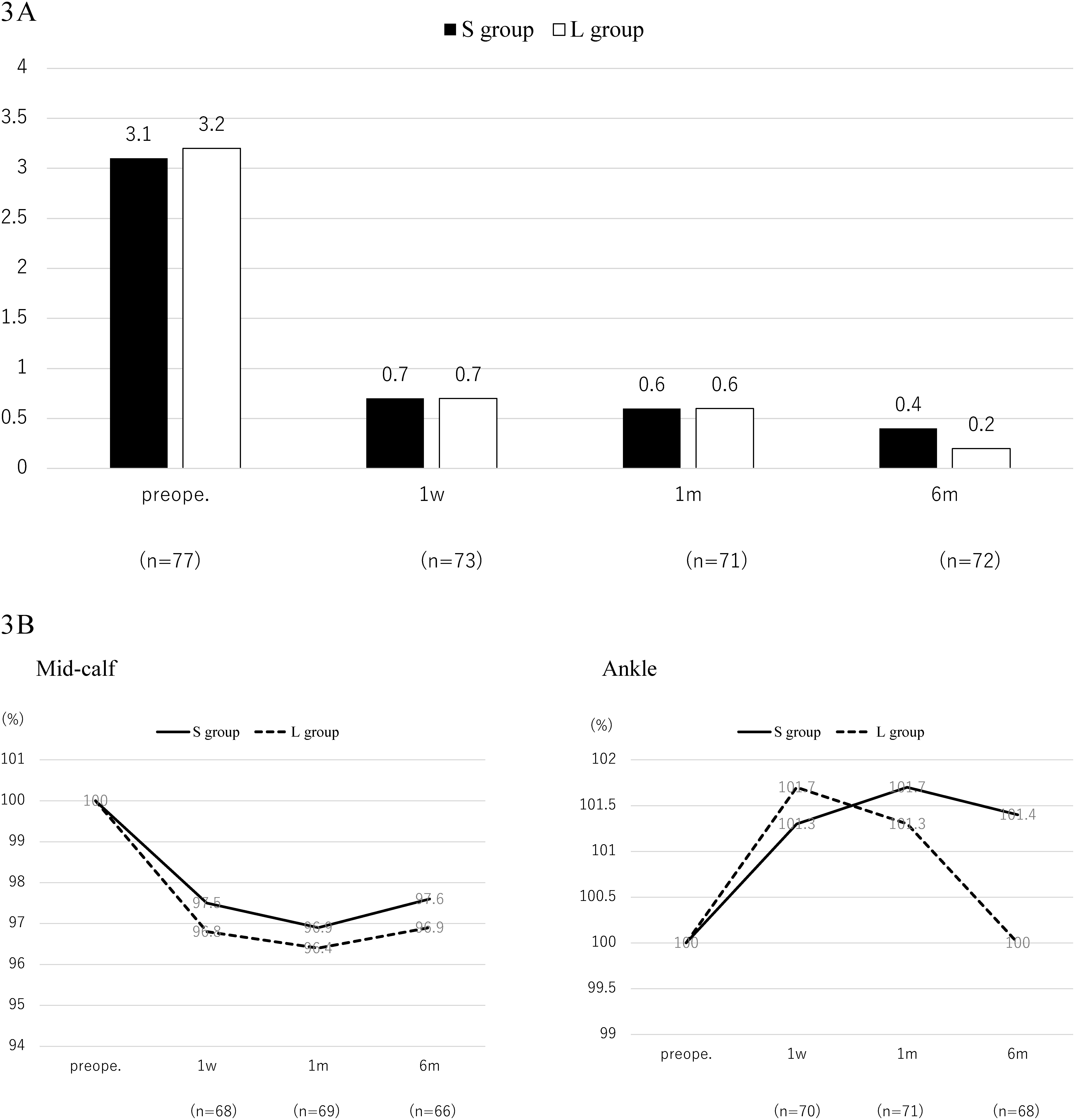
Fig. 3 Findings of secondary outcomes based on a propensity score matching analysis. (**A**) VCSS. (**B**) Leg circumference.

The VCSS did not show any significant differences between the groups at any intervals, decreasing with time in both groups (**Fig. 3A**).

VCSS: venous clinical severity score

#### Leg circumference

No significant differences were observed between the groups at any intervals in the value at the mid-calf or ankle. The mid-calf measurement decreased with time, and the curves of both groups showed a similar shape. The ankle measurement increased until 1 month, and the ankle edema persisted for longer in the S group (**Fig. 3B**).

#### Recanalization and DVT

Recanalization in the whole ablated GSV was not observed in any patients during the follow-up period.

During the follow-up period, DVT was found in three patients all belonging to the L group. One patient noticed whole-leg swelling and pain at four days after EVLA. DUS showed DVT from the calf vein to the external iliac vein, and enhanced computed tomography scan visualized pulmonary emboli. A direct oral anticoagulant was prescribed following three days’ heparin infusion, and the thrombus in the deep vein cephalad to the SFJ disappeared by 1 month after EVLA. The patient was diagnosed with protein C deficiency thereafter. The other two patients, who wore the elastic stocking for 7 days, were diagnosed with soleus vein thrombosis using DUS at 2 and 5 weeks, respectively. They underwent stab avulsion at 9 and 12 incisions, respectively, and had lived without any restriction until the diagnosis of DVT. Neither patient had any factors of thrombophilia.

#### Return to work

In the whole cohort, the median of off-work days in the S and L groups were 3 [1, 7] (n=54) and 5 [2, 8] (n=53) days, respectively. After a propensity score matching using age, sex, standing job, and CEAP clinical class as covariates, these values became 2.5 [1, 6] (n=38) and 5.5 [2, 8] (n=38) days, respectively. There was no significant difference (p=0.052) in the whole cohort, while a significant difference (p=0.018) was noted after a propensity score matching between the groups.

## Discussion

The present study showed no significant differences in any patient-reported or objective outcomes at any intervals between the S (≤2 days) and L (≥1 week) groups after overnight compression with elastic bandages after the procedure. Although ankle edema seemed to continue for longer in the S group, it had no impact on the patient-reported outcomes, such as pain relief, and did not affect the QOL, which may be more important for patients undergoing treatment for varicose disease. In addition, the most troublesome complications of EVLA (e.g., DVT or pulmonary embolism) did not occur in the S group. These results imply that subsequent compression using elastic stockings for ≥1 week following the overnight tight compression with elastic bandages after EVLA did not bring any remarkable advantages.

Postprocedural compression is widely performed for varicose vein disease after EVLA. A subcommittee of the Japanese Society of Phlebology recommended daytime compression for 1 week to 1 month following whole-day compression for several days after ETA.^4)^ All members of the subcommittee recommended the use of 20–30 mmHg of elastic stockings, and 20% and 80% of them recommended compression for 1 and 4 weeks, respectively.^4)^ We have prescribed elastic stocking use based on the Japanese guideline published in 2010,^8)^ the contents of which were almost the same as the new guideline published in 2019.^4)^ However, these recommendations were not based on solid evidence.

Several prospective studies on the optimal duration after ETA have been published,^5–7,16,17)^ and in their meta-analysis, Chou et al. summarized the results of five randomized control trials, including three studies of EVLA, one study of radiofrequency ablation (RFA), and one study of EVLA and RFA.^18)^ Patients in these studies were divided into groups receiving short compression (≤2 days) and long compression (≥1 week). The meta-analysis showed that long compression significantly reduced the postoperative pain at 1 week and improved the time of off-work. Additionally, they demonstrated that there were no significant differences in the rate of postoperative complications, leg volume, bruising scores, or QOL. Therefore, they recommended the prescription of 1-week-duration compression after ETA in routine practice.^18)^ However, two studies in the meta-analysis for pain used an 810-nm wavelength laser.^5,6)^ Another study, the data of which were not used for the meta-analysis for pain, used an 810-nm wavelength laser, which yielded a similar result.^7)^ If the 1-week compression is superior to short (≤2 days) compression based on pain relief alone, it can easily be inferred that short compression is sufficient when a high-wavelength laser is used because a high-wavelength laser results in little postoperative pain.^19,20)^

In Japan, laser systems of 980-nm and 1470-nm wavelength were launched in 2011 and 2014, respectively. Hirokawa et al. compared the clinical results of a 1470-nm diode laser and radial dual-ring fiber to those of a 980-nm diode laser and bare-tip fiber and showed that the 1470-nm diode laser and radial dual-ring fiber produced little pain at the treated vein. In addition, the occlusion rates were 100% at 12 weeks with both devices, and there was no significant difference in the postoperative complications.^9)^ Jibiki et al. also reported the superiority of a 1470-nm diode laser and radial dual-ring fiber to a 980-nm laser and a bare-tip fiber.^10)^ The present study showed no significant differences in patient-reported outcomes, including the VAS and use of analgesics, between the S and L groups, suggesting that our hypothesis is correct.

Another important point in the treatment for varicose vein disease is not to hamper the early return to work of patients with a regular job. In the present study, the off-work period in the S group was shorter than that of the L group; this finding differed from the results of the meta-analysis.^18)^ Since most of the patients applied for time off-work before admission, the off-work period was not markedly affected by the duration of compression in the present study, which simply demonstrated that short compression never delayed the restart of the job.

This study is associated with several limitations. First, the protocol was not registered before the start of the study. Second, it was a nonrandomized study. Complete randomization was difficult because compulsory grouping might have hampered the cooperation of the patients, and compliance could not be held to be high. Only 2.0% of patients, who selected the L group and underwent EVLA, discarded their stockings prior to the completion of 1 week of follow-up in the present study. This rate was much lower in comparison to other reports (8.5% and 8.8%).^5,6)^ Third, phlebectomy simultaneously performed with EVLA might have affected the patient-reported outcomes. It was confirmed that the number of incisions did not affect postoperative pain (data not shown). However, the skin condition of the incision (i.e., the presence of inflammation or induration) might affect the postoperative pain. Fourth, the patient-reported outcomes in the earlier period (prior to 1 week) were not checked. The difference in the pain between the groups might disappear at 1 week. The present study showed that there was no difference in the use of analgesics within 1 week after discharge between the groups, suggesting that the pain was not severe, even if the VAS might be higher in the earlier period. Although there seemed to be some discrepancy between the results of VAS and VCSS including the evaluation of pain, this discrepancy may be derived from the difference of evaluation methods. Fifth, an elastic stocking of 20 mmHg at the ankle was used for the additional compression. The pressure is lower than that of stocking used in the other studies.^5,6,16,17)^ The outcomes might be affected by the pressure of the elastic stocking.

## Conclusion

There were no significant differences in the patient-reported or objective outcomes between the S (≤2 days) and L (≥1 week) groups. Wearing elastic stockings for ≥1 week after the overnight compression with elastic bandages after EVLA for primary varicosity, using a 1470-nm diode dual-ring radial laser fiber, brings few advantages, suggesting that a short (≤2 days) duration of additional compression might be sufficient.
